# Women's Satisfaction of Maternity Care in Nepal and Its Correlation with Intended Future Utilization

**DOI:** 10.1155/2015/783050

**Published:** 2015-11-08

**Authors:** Yuba Raj Paudel, Suresh Mehata, Deepak Paudel, Maureen Dariang, Krishna Kumar Aryal, Pradeep Poudel, Stuart King, Sarah Barnett

**Affiliations:** ^1^Nepal Health Sector Support Program, Ministry of Health and Population, Kathmandu 44600, Nepal; ^2^Department for International Development in Nepal, Country Office, Ekantakuna Road, Lalitpur 44600, Nepal; ^3^Nepal Health Research Council, Ministry of Health and Population, Kathmandu 44600, Nepal; ^4^Options Consultancy Services Limited, Devon House, 58 St. Katharine's Way, London E1W1LB, UK

## Abstract

The impact of rapid increase in institutional birth rate in Nepal on women's satisfaction and planned future utilization of services is less well known. This study aimed to measure women's satisfaction with maternity care and its correlation with intended future utilisation. Data came from a nationally representative facility-based survey conducted across 13 districts in Nepal and included client exit interviews with 447 women who had either recently delivered or had experienced complications. An eight-item quality of care instrument was used to measure client satisfaction. Multivariate probit model was used to assess the attribution of different elements of client satisfaction with intended future utilization of services. Respondents were most likely to suggest maintaining clean/hygienic health facilities (42%), increased bed provision (26%), free services (24%), more helpful behaviour by health workers (18%), and better privacy (9%). Satisfaction with the information received showed a strong correlation with the politeness of staff, involvement in decision making, and overall satisfaction with the care received. Satisfaction with waiting time (*p* = 0.035), information received (*p* = 0.02), and overall care in the maternity care (<0.001) showed strong associations with willingness to return to facility. The findings suggest improving physical environment and interpersonal communication skills of service providers and reducing waiting time for improving client satisfaction and intention to return to the health facility.

## 1. Introduction

The Government of Nepal has promoted institutional births through the expansion of birthing centers in existing peripheral health institutions and the availability of 24-hour comprehensive emergency obstetric care at hospitals [1]. In addition, the Ministry of Health and Population (MoHP) introduced maternity incentives to reduce financial barriers to accessing institutional births in 2005, which evolved into free maternity care and transport incentives (the Aama program) in 2009 [2]. As a result of this demand generation and service expansion, the institutional birth rate tripled from 18% in 2006 to 55% in 2014 [3–6]. However, increasing the access and utilization of health services is unlikely to bring improved health outcomes unless services meet benchmarks for good quality [7]. The midterm review of current Nepal Health Sector Program (NHSP II) acknowledged that attention to date has focused on improving access to care, and, although this needs to continue, more attention on quality of care is required as a matter of priority [8]. Furthermore, quality of care is also a central focus in the National Health Policy 2014 [9].

Women's experience and satisfaction are an important element of quality of maternity care [10, 11]. Satisfaction is a complex and multidimensional concept embracing structure, process, and outcome of care [12, 13]. The literature suggests that factors such as women's participation in decision making during pregnancy and childbirth [14, 15], women's sense of control, both internal and external, over the whole process [13, 14], client-provider relationships [16], respectful care [7], and the physical environment of the maternity ward [15] are significant factors associated with women's satisfaction and future utilization of health services. A systematic review highlights the importance of staff attitude and respectful behaviour over pain management or sociodemographic factors on maternity client satisfaction [17]. Hence, to understand service user's perception of quality service, and interaction among different elements of quality of care, it is necessary to study correlation of satisfaction with these elements. Administrators and managers can use such information to improve quality score in a cost-effective way.

Client satisfaction measures the ability of services to meet consumers' expectations [18], and is an important determinant of the choice of health facility and of future utilization of services [19–22]. Satisfied clients will be more likely to return in the future and recommend the institution to their relatives/friends [23, 24]. A study conducted in outpatient setting to investigate association of patient satisfaction with return behaviour concluded that many of the standard elements of quality of care have a very less effect on return behaviour, whereas time and attention paid to health care users were the strongest predictor of returning to a health institution [24]. However, to our knowledge, no studies have examined the association between the likelihood of returning to facility with maternity clients' satisfaction in Nepal.

Increased institutional births are being successfully promoted in Nepal; however, the impact of rapid increases in utilisation on quality of care, women's experience, and client satisfaction is less well known. This paper aims to measure client satisfaction with key elements of quality of care and study the correlation between key quality measures and future utilisation. Understanding women's views and experiences provides an important insight for managers and policy makers to change practices to effectively address their needs and expectations and benefit future clients [25].

## 2. Methods

### 2.1. Data Source and Sampling

This paper used data from a nationally representative cross-sectional facility-based survey conducted by some of the authors: Service Tracking Survey (STS) 2013 [26]. Three questionnaires were administered in the survey: facility assessment, exit interviews with maternity clients, and exit interviews with outpatients. The survey provided national estimates for key reproductive, maternal, neonatal, and child health indicators related to availability, readiness, and quality of care. The detailed methodology is presented in the STS 2013 final report [26]. Briefly, a two-stage sampling design was adopted to select health facilities. In the first stage, five districts from Terai, five districts from hill, and 3 districts from mountain were selected considering one district (primary sampling unit) that was randomly selected from 13 subregions of Nepal. In the second stage, all district hospitals and Primary Health Care Centres (PHCCs) from selected districts were included, and sub/health posts (S/HPs) were selected using equal probability selection method (EPSEM). The selected health facilities included 17 public hospitals, 39 PHCCs, 100 HPs, and 86 SHPs. A total of 447 exit interviews were conducted with women who had either delivered recently or had experienced obstetric complications (87% in hospitals, 8% in PHCCs, 4% in HPs, and less than 1% in SHPs). Due to low caseload and the short data collection time period, fewer clients were interviewed in HPs and SHPs.

### 2.2. Data Collection and Quality Assurance

Data collection was carried out between July and August 2013. Training Manual, Survey Field Manual, and Data Entry Manual were produced and used throughout the training, data collection, and data entry to ensure quality and consistency. Enumerators had a five-day training, focusing on objectives, approach, survey instruments, ethical issues, reporting, and other operational issues. Supervision and monitoring visits to the survey sites were made soon after survey started to identify and rectify any problems early on. Completed questionnaires were checked by the supervisors in the district before sending them to the central office for data entry. Feedback was provided to the enumerators during data collection.

### 2.3. Data Cleaning, Coding, and Entry

Completed questionnaires were checked for completeness, consistency of data, and the presence of outliers before data entry. Any suspect data were cross-checked against hard copies of completed questionnaires. The databases were developed in CSPro 5.0. The databases were pretested before data entry start and any errors were eliminated.

### 2.4. Measures

#### 2.4.1. Client Satisfaction

Client satisfaction was measured using an eight-item instrument. The items covered several key dimensions of client satisfaction: accessibility (one question), interpersonal communication (two questions), physical environment (two questions), clinical care (two questions), and decision making (one question). The 8 items of quality of care showed a high internal consistency (Cronbach's alpha = 0.74) to measure client satisfaction. The responses were marked using a five-point Likert scale [27]: (1) fully dissatisfied, (2) unsatisfied, (3) neutral, (4) satisfied, and (5) fully satisfied. The survey measured the likelihood of visiting the facility again with the question “if you are willing to have another baby, would you like to visit this facility for childbirth?”

#### 2.4.2. Data Analysis

We acknowledged the weighing of the data, the approximate stratification, and the two-level clustering while computing statistical tests, using the survey functions of STATA 12 SE Version.

**Figure 1:**
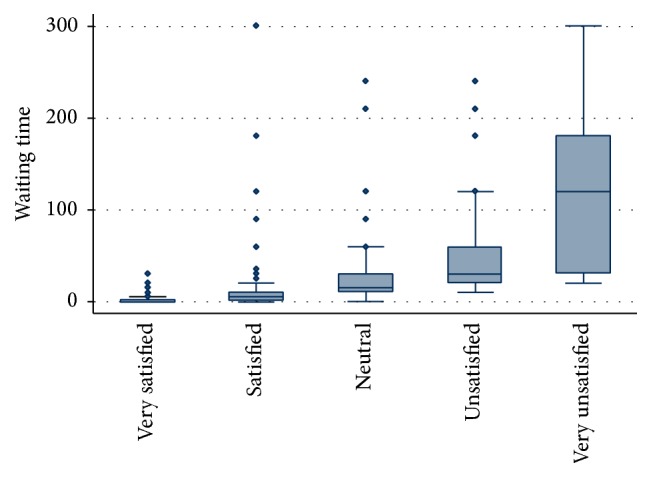
Waiting time (minutes) according to level of satisfaction with waiting time (*N* = 447).

**Figure 2:**
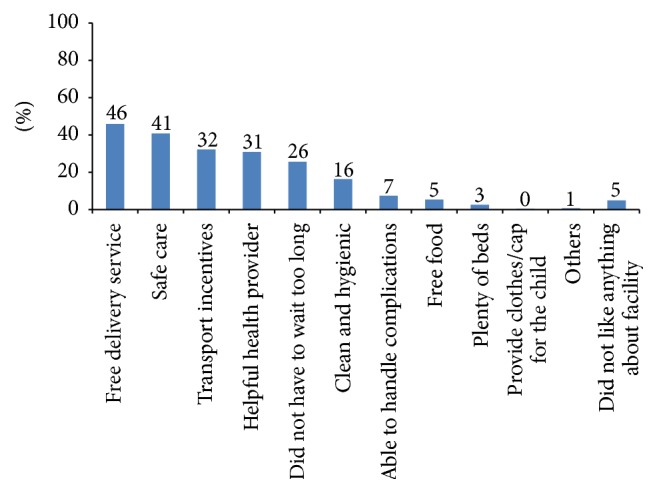
Maternity clients' likes about delivery care (*N* = 447).

**Figure 3:**
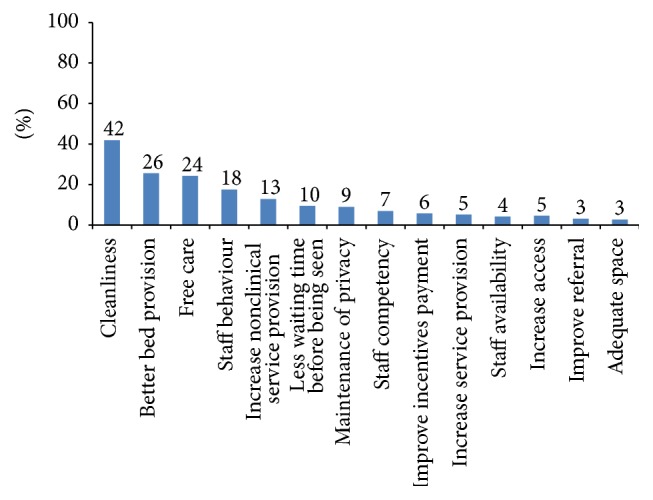
Maternity clients' recommendations (*N* = 447).

**Figure 4:**
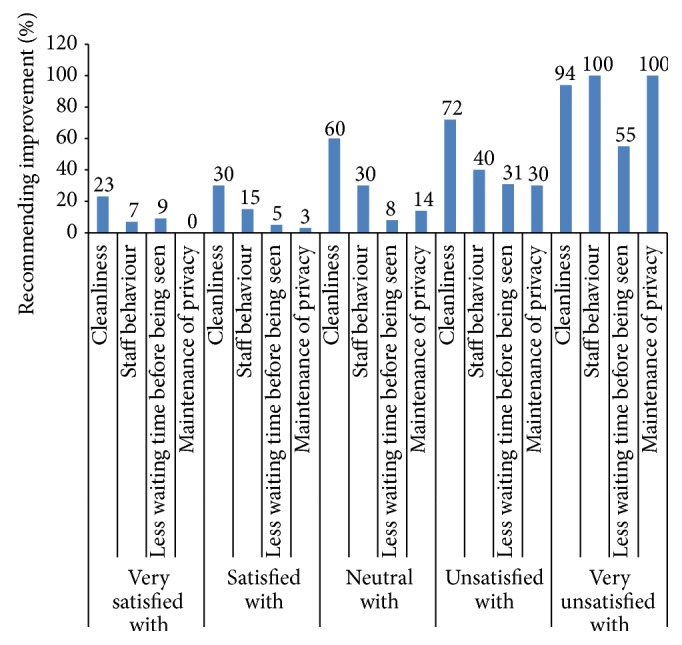
Maternity clients' recommendations according to level of satisfaction (*N* = 447).

The sample weight was used during the descriptive bivariate and multivariate analysis. Descriptive analysis was carried out for all dimensions of clients' satisfaction (Table 3). Pearson's correlation was calculated between levels of satisfaction with waiting time, information received, provider competency, politeness of staff, involvement in decision making, cleanliness of facility, privacy, overall care received at the facility, and intended future use of services (Table 2). A correlation coefficient of ≥0.3 is considered to be a strong correlation for this analysis. A multivariate probit regression model was used to investigate factors associated with intention of future use of services (Table 4). The multivariate model included 8 items of client satisfaction. Level of satisfaction according to waiting time was investigated and presented in a box plot (Figure 1). Maternity clients' likes and recommendations to physical environment, staff behaviour, and facilities were calculated as percentage of total respondents (Figures 2 and 3). Furthermore, client's recommendations to improve various attributes (waiting time, cleanliness, privacy, and staff behaviour) according to their level of satisfaction to each item were also investigated (Figure 4).

## 3. Results

Table 1 depicts sociodemographic characteristics and accessibility factors for maternity clients. Among 447 respondents, nearly two-thirds were 20–29 years old (67%) and more than one-third were Brahmin/Chhetri (37%). Nearly half (46%) of the total sample had completed secondary education. It took less than 30 minutes for around one-third of the respondents (37%) to reach the health facility, while it took more than an hour to reach health facility for more than a quarter (26%) of respondents.

The percentages of clients satisfied with individual elements of quality of care are presented in Table 2. Most of them were satisfied with (very satisfied and satisfied) care received at the facility (86%), provider's skills (85%), politeness of staff (83%), waiting time (80%), involvement in decision making (77%), cleanliness (70%), information received (69%), and assured confidentiality (67%). Mean satisfaction score was the highest for level of skill of service provider (4.0) and was the lowest for cleanliness of the facilities (3.4).

Table 3 shows the correlation between the various elements of client satisfaction. Satisfaction with information received showed a strong correlation with politeness of staff, involvement in decision making, and satisfaction about care at facility. Likewise, satisfaction with skill of service provider also showed a strong correlation with politeness of staff and satisfaction with care received. Furthermore, a strong correlation was observed between future use of services and the overall care received at the facility.

Determinants for willingness to visit the facility again are shown in Table 4. Just more than half of the respondents (56%) reported that they were willing to visit the facility again (data not shown). Multivariate analysis revealed that satisfaction with waiting time (coef.: 0.47; 95% CI: 0.03–0.90), information received at the facility (coef.: 0.64; 95% CI: 0.09–1.19), and satisfaction with overall care at facility (coef.: 1.03; 95% CI: 0.55–1.50) were positively associated with willingness to visit the facility again. No significant association was observed with willingness to visit the facility again and politeness of staff, involvement in decision making, cleanliness of facility, and privacy at facility.

Figure 1 describes satisfaction with waiting time versus waiting time duration at the facility, and study revealed that the likelihood of dissatisfaction with waiting time is increased with increase in waiting time at the facility. The significant difference in waiting time was observed by level of satisfaction; the lowest waiting time was observed for those who were very satisfied (mean: 2 minutes and median = 0 minutes) compared to those who were very unsatisfied (mean: 144 minutes and median: 120 minutes).

Maternity clients were asked what they liked or disliked about the childbirth care they had received. Most commonly, clients liked the provision of free delivery services (46%); safe care (41%); transportation incentives (36%); the helpful attitude of health workers; short waiting times; and the clean and hygienic conditions of health facilities (Figure 2). The most common dislikes reported by maternity clients were a lack of cleanliness (22%), a scarcity of beds and bed linen (21%), and a lack of privacy (9%).

Figure 3 presents major recommendations made by maternity clients to improve services. Most of them suggested maintaining clean/hygienic health facilities (42%), better bed provision (26%), improvement/continuity of free services (24%), more helpful behaviour from health workers (18%), less waiting time (10%), and better privacy at the health facilities (9%). About 17% of maternity clients responded that everything was good in the facility and required no improvement.

Figure 4 shows recommendations of maternity clients by level of satisfaction to cleanliness, staff behavior, waiting time, and privacy. More than 9 in 10 (94%) of clients who were very unsatisfied with cleanliness recommended improving the cleanliness of the facility. More than half of clients who were satisfied or very satisfied with cleanliness also recommended improving cleanliness of the facilities. All of the clients who were very unsatisfied with staff behavior and privacy recommended improving staff behaviour, and privacy, respectively, in the facility. More than half of the clients (55%) who were very unsatisfied with waiting time made recommendation to reduce waiting time between arriving at the facility and being seen by service provider.

## 4. Discussion

This study measured client satisfaction using 8 items of quality of care. Mean satisfaction score was the highest for level of skill of service providers. On one hand, clients may not be able to differentiate dimensions of competence and incompetence. On the other hand, clients may relate provider skill with politeness and good communication skill of service providers as shown by correlation matrix (Table 2). Previous studies have also found that frequency of explanations [15], skillful interactions, and responsiveness of service provider to client's need [28, 29] were strongly associated with satisfaction with maternity care received. Furthermore, quality of care might have different meaning to different individuals [10]. Relational component could be more important to some clients compared to technical competency of service providers [7].

Cleanliness of the facility was a major concern among both satisfied and unsatisfied clients. Just more than two-thirds of the clients were satisfied or very satisfied with cleanliness. While a similar proportion of clients were satisfied or very satisfied with cleanliness in service tracking survey of 2012 [30], higher dissatisfaction with cleanliness of maternity facilities was shown in studies conducted in Malawi [21] and Kenya [20] in comparison to current study. Since cleanliness is easily discernible to clients in comparison to other aspects of quality of care, this could have resulted in lower satisfaction with cleanliness. However, poor cleanliness in maternity wards has been reported in previous studies conducted in Nepal and elsewhere [20, 21, 31]. A study that used pattern approach to studying satisfaction to maternity care showed that women who were unsatisfied with physical environment were more likely to be educated [29]; since more than two-thirds of clients in current study had secondary or higher education, cleanliness could have been pointed out clearly. It is interesting to note that almost a quarter of clients who were very satisfied with cleanliness also expressed recommendation to improve cleanliness of the facilities (Figure 4).

Satisfaction with information received showed a strong correlation with politeness of service provider, skill of service provider, and satisfaction toward the care received. This finding suggests that women want to be well informed about the process and outcome of childbirth and most likely relate it to competency of service provider. A good communication between provider and clients is highly valued by maternity clients [25]. Although research has shown that cognitive and emotional support for women during labour is beneficial for well-being of women and newborn by reducing duration of labour and possibility of postpartum depression [32], many studies have highlighted that maternity experience in medical setting has been dominated by professionals. Health workers share very less information with mothers about childbirth process, let alone participating them in decision making about childbirth [25, 33]. Furthermore, although clients expect service providers to have knowledge and technical competency, their satisfaction is mainly determined by behaviour, communication skill of service providers, and amount of time spent in interaction [17, 34]. Hence, maternity health care need to be restructured in a way to cater to the multidimensional needs of women during childbirth.

Only two-thirds of clients were satisfied or very satisfied with privacy in the facility. A study conducted in a maternity health centre from Malawi found that, despite being treated politely, lack of auditory and visual privacy led women to not using a maternity facility [21]. Hence provisions to ensure privacy in health facilities are warranted.

Similar to the findings from other studies [18, 34, 35], the current study found that client satisfaction decreased with higher waiting time. Being seen only after 2 hours of arriving at facility could have brought feeling of being ignored at the health facility and brought dissatisfaction. Another possible explanation for dissatisfaction with longer waiting time could be due to less time to talk with service providers owing to overcrowded facility, despite having to wait for long time. Furthermore, a study conducted in Ethiopia found that delay in receiving care once women had reached the maternity hospital was mainly due to operational issues such as “shortage of medicines, blood, equipment, or to the absence of qualified/competent staff, poor organization of care or combination of all” [36]. Although current study could not investigate what caused longer waiting time, it is likely that poor organization and readiness of care could have resulted in longer waiting time since Jahn et al. found that conduction of caesarean section took an average of 4.5 hours (range of 40 minutes to 11 hours) in rural Nepal once the decision to operate has been made [37]. Longer waiting time was reported to be associated with the worst outcomes among women who experienced similar childbirth complications [36]. Increased demand with staff shortage is likely to result in overcrowded facilities, longer waiting time, poor behaviour from overburdened staff, and shortage of supplies [38], which are often interrelated. Since childbirth is a stressful event, women want quick response when they need a support, while a longer waiting time causes frustration/dissatisfaction [34].

Current study found an association of willingness to return to the facility with satisfaction to waiting time, information received, and overall care in the facility. These results are in concert with findings from other recent studies [23, 34, 39–41]. A study from the US also showed that reducing waiting-room wait time in a primary-care practice significantly improved patient satisfaction and willingness to refer relatives/friends to the facility [41]. Willingness to refer friends/relatives was treated as a proxy measure for overall satisfaction and willingness to return to the primary care. A previous study concluded that service providers need to assess expectation of clients regarding realistic waiting time in order to meet their expectations and improve satisfaction [34]. Reduced intention to return to health facility among those who had to wait for long time could be attributed to poor outcomes among mothers having to wait longer before being seen [36].

Consistent with findings from previous studies [23, 40], information received from service providers, and patient satisfaction have been shown to be a strong correlate of return behaviour in the current study. Garman et al. showed that satisfied clients were more likely to return to the hospital [24]. The clients who returned for subsequent health care were more likely to have received adequate information and attention from service providers. The time and attention provided to the patients and their families counted a lot in increasing likelihood for subsequent visit to the same institution. Hence, high quality patient-clinician relationship is instrumental for client satisfaction. Similarly, Al-Mailam studied satisfaction of hospital care and found that satisfaction to overall care was significantly associated with satisfaction to nursing care. And satisfaction to nursing care showed a strong correlation with intention to return to the hospital [23]. Studies show that if nurses are satisfied with their own jobs, they will behave with clients in a respectful manner [23, 42]. Since most of the care in maternity care is associated with nursing care, behaviour of nurses is likely to determine overall satisfaction and probability of returning in the future.

The findings of this study need to be interpreted in the light of some limitations. Since the majority of women were interviewed within 24 hours of birth in institution, there is a possibility of women being less critical due to the joy of childbirth and overlooking negative experiences due to a phenomenon called halo effect [43]. Further, women of early postnatal period feel difficulty to report negative experiences of childbirth if the child is healthy [44]. In addition, being interviewed within institutional setting might have caused women to response in a positive way. Although other studies have used willingness to recommend the facility as a measure of satisfaction, this study only examined willingness to return to facility. However, researchers have treated willingness to recommend the health facility to friends/relatives as a proxy measure for willingness to return to health facility [41]. Since only few maternity clients (5% of total sample) could be interviewed from sub/health posts, the findings from this study might be closer to the scenario of PHCCs and hospitals of Nepal. We measured intention to return to the health facility for next childbirth which could be different to their actual behaviour. First-time mothers, less educated [29] ones, or who gave childbirth at institutions for the first time might have different perception of quality of care compared to mothers who are educated and who have a previous experience of giving birth at institution.

The findings of this study have implications for policy, maternity care practice, and future research. With increasing focus on institutional birth with skilled birth attendants there is a fear that biomedical interventions overshadow the psychosocial model of care for women [33, 45]. Hence women's expectations need to be understood and addressed upon. Being treated with kindness and meeting their expectations increase women's satisfaction of childbirth experience [25, 46]. Cleanliness of maternity facilities and adequate beds and bed linens need to be ensured and privacy needs to be maintained. Reducing waiting time and providing adequate information are critical for increasing the likelihood to return in the future. Altogether, a renewed focus needs to be given to provide women with full information without having to wait for too long. They need to be provided with opportunity to ask questions and allowed to be involved in decision making. Further qualitative studies examining expectation of clients and satisfaction with self (internal control) need to be undertaken to better understand women's experiences. Response time (duration between calling for service and receiving service) need to be included as measures of satisfaction in future studies.

## 5. Conclusion

Mean satisfaction score was the highest for skill of service providers and the lowest for cleanliness of facilities. Satisfaction with information received was strongly correlated with politeness of staff, involvement in decision making, and satisfaction with overall care at facility. Willingness to return to facility showed a strong association with information received, waiting time, and overall care at facility. Hence, the measures to improve client experience of maternity care in Nepal should focus on improvement in physical environment along with improving attitude and communication skill of service providers with prompt response.

## Figures and Tables

**Table 1:** Sociodemographic characteristics and geographical/accessibility factors of maternity clients (*N* = 447).

Variables	*N*	%
Sociodemographic characteristics		
Age (years)		
<20	109	24.38
20–29	300	67.11
≥30	38	8.50
Parity		
Primigravida	263	58.84
Multigravida	184	41.16
Education status		
Never attended school	92	20.58
Primary education	57	12.75
Secondary education	204	45.64
Further education	94	21.03
Caste/ethnicity		
Brahmin/Chhetri	164	36.69
Terai/Madhesi other castes	91	20.36
Dalits	68	15.21
Newar	15	3.36
Janajati	96	21.48
Muslim	13	2.91
Geographic/assessable factors		
Ecological zone		
Mountain	38	8.50
Hill	136	30.43
Terai	273	61.07
Place of residence		
Urban	346	77.40
Rural	101	22.60
Reaching time (minutes)		
<30	167	37.36
30–59	160	35.79
≥60	120	26.85

**Table 2:** Percentage of the mothers by level of satisfaction with perinatal care (*N* = 447).

Dimension	Very satisfied	Satisfied	Mean satisfaction score^*∗*^	Standard deviation
Accessibility				
Waiting time	27.9	52.5	3.9	0.95
Interpersonal communication aspects				
Information received	11.2	57.7	3.7	0.70
Politeness of staff	12.8	69.9	3.9	0.64
Physical environment				
Assurance of confidentiality	4.0	62.7	3.6	0.73
Cleanliness of facility	7.2	62.4	3.4	0.99
Decision making				
Involvement in decision making	8.9	68.3	3.8	0.65
Technical aspect				
Level of skill of provider	17.3	67.6	4.0	0.62
Care at facility	12.9	72.8	3.9	0.60

^*∗*^The satisfaction score was constructed by giving scores: fully satisfied = 5; satisfied = 4; neutral = 3; dissatisfied = 2; and fully dissatisfied = 1.

**Table 3:** Correlation matrix (*N* = 447).

	Waiting time	Information received	Level of skill of provider	Politeness of staff	Involvement in decision making	Cleanliness of facility	Level of privacy	Care received at facility	Intention for future use of services
Satisfaction with									
Waiting time	1.00								
Information received	0.23	1.00							
Level of skill of provider	0.28	0.41	1.00						
Politeness of staff	0.31	0.36	0.49	1.00					
Involvement in decision making	0.19	0.43	0.30	0.38	1.00				
Cleanliness of facility	0.19	0.25	0.20	0.15	0.19	1.00			
Level of privacy	0.11	0.33	0.23	0.26	0.24	0.27	1.00		
Care received at facility	0.24	0.40	0.43	0.43	0.31	0.32	0.38	1.00	
Intention for future use of services	0.19	0.14	0.13	0.23	0.15	0.08	0.09	0.38	1.00

**Table 4:** Determinants of willingness to return to facility (*N* = 447).

	Linearized				95% confidence interval	
	Coef.	Std. err.	*z*	*p*	Lower	Upper
Waiting time	0.47	0.22	2.11	0.035	0.03	0.90
Information received	0.64	0.28	2.29	0.022	0.09	1.19
Politeness of staff	0.00	0.26	−0.02	0.986	−0.51	0.50
Privacy at facility	−0.39	0.24	−1.64	0.100	−0.86	0.08
Cleanliness of facility	−0.18	0.20	−0.91	0.365	−0.56	0.21
Involvement in decision making	0.15	0.22	0.68	0.500	−0.29	0.59
Level of skill of provider	−0.28	0.27	−1.05	0.292	−0.81	0.24
Overall care at facility	1.03	0.24	4.24	<0.001	0.55	1.50
